# Unraveling the Spatiotemporal Human Pluripotency in Embryonic Development

**DOI:** 10.3389/fcell.2021.676998

**Published:** 2021-06-23

**Authors:** Daniela Ávila-González, Wendy Portillo, Guadalupe García-López, Anayansi Molina-Hernández, Néstor E. Díaz-Martínez, Néstor F. Díaz

**Affiliations:** ^1^Instituto de Neurobiología, Universidad Nacional Autónoma de México, Juriquilla, Mexico; ^2^Instituto Nacional de Perinatología, Mexico City, Mexico; ^3^Biotecnología Médica y Farmacéutica, Centro de Investigación y Asistencia en Tecnología y Diseño del Estado de Jalisco, Guadalajara, Mexico

**Keywords:** naïve, primed, amnion, formative, synthetic biology

## Abstract

There have been significant advances in understanding human embryogenesis using human pluripotent stem cells (hPSCs) in conventional monolayer and 3D self-organized cultures. Thus, *in vitro* models have contributed to elucidate the molecular mechanisms for specification and differentiation during development. However, the molecular and functional spectrum of human pluripotency (i.e., intermediate states, pluripotency subtypes and regionalization) is still not fully understood. This review describes the mechanisms that establish and maintain pluripotency in human embryos and their differences with mouse embryos. Further, it describes a new pluripotent state representing a transition between naïve and primed pluripotency. This review also presents the data that divide pluripotency into substates expressing epiblast regionalization and amnion specification as well as primordial germ cells in primates. Finally, this work analyzes the amnion’s relevance as an “signaling center” for regionalization before the onset of gastrulation.

## Introduction

Contemporary developmental biology has the challenge of deciphering the molecular bases that regulate processes during development and how these elements interact to establish patterns and construct a functional organism. Before gastrulation, embryonic cells are in a pluripotent state, which confers them the ability to differentiate into all the cells derived from the germ layers. Although pluripotency is ephemeral during development, it also constitutes a dynamic phase with molecular changes representing the epiblast’s transition before, during and after implantation (Nakamura et al., 2016). Hence, the derivation and continuous maintenance of embryonic stem cell (ESC) lines derived from preimplanted embryos of different species, but especially of the mouse, open a new window to identify distinct pluripotent states *in vitro* with specific molecular profiles and development potentials.

Pluripotency can also be spatially regionalized, suggesting that it is more than a transitory identity to maintain the undifferentiated epiblast before the onset of morphogenesis. Thus, there may also be substrates in the same period, which probably depend on intrinsic and extrinsic signals—depending on their position—to acquire the competence to differentiate in the anterior-posterior structures (Peng et al., 2019). Although the knowledge on pluripotency focuses on the mouse embryo and its *in vitro* counterparts, the human pluripotency spectrum could be more “heterogenous” due to differences with murine development. For example, in primates the epiblast coexists with extraembryonic lineages (the amniotic epithelium and extraembryonic mesoderm (EXM) before the gastrulation stage (Boroviak and Nichols, 2017). Thence, these cellular interactions could imply pluripotency states still not characterized or present in the murine model compared with the human model. Analyzing the molecular and functional spectrum of human pluripotency (intermediate states, pluripotency subtypes, and regionalization) is essential to understand the initiation of human gastrulation and morphogenesis.

## Specification of the Human Epiblast and Its Differences With the Mouse

The endoderm, ectoderm and mesoderm arise from the epiblast, whereas the placenta differentiates from the trophectoderm (TE), and the primitive endoderm (PE) generates the yolk sac. Later, the epiblast contains the necessary information to initiate morphogenesis and embryogenesis once implantation occurs (Hermitte and Chazaud, 2014). The epiblast and PE originate from the inner cell mass (ICM) during the mouse’s second lineage specification.

**FIGURE 1:**
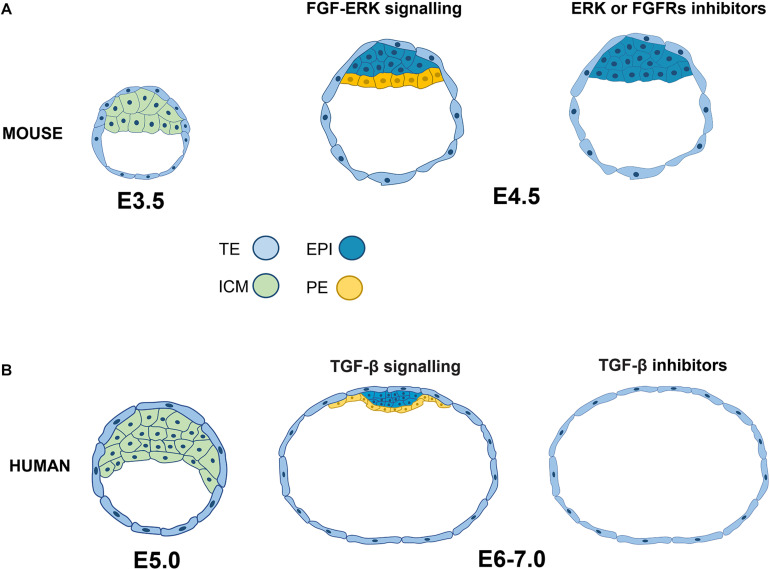
Signaling pathways related to the specification and segregation of pluripotent epiblast lineages in mouse and human. **(A)** In the mouse embryo at 3.5 days post-fertilization (E3.5), the ICM coexpresses NANOG and GATA6. By day 4.5, the ICM segregates into NANOG^+^ EPI and GATA6^+^ PE. EPI specification occurs in cells with low levels or suppression of the FGF-ERK pathway. **(B)** In human embryos, there is also colocalization of NANOG and GATA before lineage segregation, but the inhibition of FGF-ERK does not induce alterations in the specification of EPI (NANOG^+^, OCT4^+^) or PE (GATA4^+^). In contrast, blocking TGF-β signaling promotes the deficiency of both NANOG and SOX17, so this pathway is essential for the segregation of EPI and EP in the human embryo. ICM, inner cell mass; EPI, epiblast; PE, primitive endoderm; TE, trophoectoderm.

At a stage of 3.5 days post-fertilization (E3.5), the ICM coexpresses NANOG and GATA6, and, at E4.5, NANOG^+^ (epiblast) and GATA6^+^ (PE) populations are segregated by FGF4 signaling (Yamanaka et al., 2010). Thus, the FGF receptor 1 (FGFR1)^+^ cells together with the autocrine FGF4 signaling commit to the epiblast, while paracrine FGF4 signaling through FGFR1 and FGFR2 derive the PE lineage. The relatively low ERK levels in FGFR1^+^ cells trigger ETV and SPRYs (FGF targets), which induce negative feedback with ERK, increasing the NANOG expression and subsequent epiblast identity. In contrast, FGF-ERK signaling in FGFR1^+^/FGFR2^+^ cells activates alternative targets, such as DUSP, to reduce NANOG expression and GATA6 is maintained in the prospective PE (Kang et al., 2017). FGF signaling suppression through MEK inhibitor PD0325901 (PD032) or FGFR inhibitor PD173074 induces conversion into epiblast and no specification into the PE at the morula in murine (Figure 1A) and bovine cells (McLean et al., 2014; Bessonnard et al., 2017). Consistent with reducing MEK activity to establish naïve pluripotency (see later) in the preimplantanted epiblast, PD032 is an essential component of the cocktail to derive and maintain naïve mouse embryonic stem cells (mESCs) (Nichols et al., 2009). Like in the mouse, the ICM in human blastocyst at 6 days after *in vitro* fertilization (IVF), showed GATA6 and NANOG colocalization before the appearance of PE and epiblast at IVF day 7 (Roode et al., 2012). However, MEK or FGFR inhibitor treatment does not change NANOG, OCT4, or GATA4^+^ cell numbers, suggesting that FGF-MEK signaling is not required in human embryos for the second lineage specification (Roode et al., 2012). Therefore, although the segregation of the ICM into the PE and epiblast is similar in both species, their signaling pathways contrast. These data suggest that specific molecular and cellular mechanisms induce the initiation and maintenance of epiblast pluripotency in humans.

## The Human Pluripotency Signaling Pathways

### TGF-B Signaling in the Epiblast and Human Pluripotent Stem Cells (hPSC)

Unlike FGF signaling, the TGF-β pathway is crucial to defining and maintaining the human epiblast’s identity. TDGF1, TGFBR1, and ALK1 receptors, NODAL and GDF3 ligands and SMAD2/SMAD4 transcription factors are expressed in the human epiblast. Interestingly, SB431542 (SB) treatment disrupts the TGF-β/Activin/Nodal pathway through inhibition of ALK5, ALK4, and ALK7 receptors, which induces NANOG and SOX17 downregulation in human embryos at days 6–7, indicating that the TGF-β pathway is relevant for epiblast and PE segregation (Figure 1B). Moreover, SMAD2/SMAD5, TGFB1 and the negative regulator TGFBR3 are highly enriched in the trophectoderm (TE), suggesting the importance of this pathway in the first lineage specification (Blakeley et al., 2015).

Accordingly, the TGF-β/Activin/Nodal pathway is essential to maintain *in vitro* human pluripotency. hESCs cultured with iMEF conditioned medium (hESC-iMEFCM) or medium supplemented with Activin A (10 ng/mL) display SMAD 2/3 nuclear phosphorylation and have high OCT4 levels; while in differentiating cells growing in non-conditioned and non-supplemented media, the SMAD proteins decrease and their location is cytoplasmic (James et al., 2005; Vallier et al., 2005). hESCs treated with Activin A inhibitor (follistatin) lack the surface antigen TRA-1-60 expression, whereas the ALK4/5/7 receptors’ competitive inhibition by SB compromises their undifferentiated state and decreases NANOG and OCT4 levels as well as TRA-1-60 expression (James et al., 2005; Vallier et al., 2005). In contrast, SB treatment in mESCs induces low phospho-SMAD2 levels but does not affect the OCT4 level (James et al., 2005). Also, SB treatment does not affect NANOG^+^, OCT4^+^, or even SOX17^+^ cells in mouse blastocyst, contrasting with its human counterpart (Blakeley et al., 2015). Hence, the TGF-β/Activin/Nodal pathway activated through ALK receptors and mediated by SMAD2/3 is essential for hESCs and human pluripotency during development compared with preimplantation mouse embryo and mESCs. However, this pathway is not enough to support human pluripotency. Indeed, Activin or Nodal with FGF2 can maintain the pluripotent markers’ expression for a long time (more than ten passages) in the absence of feeders (Vallier et al., 2005).

A modified culture media supplemented with Activin A and small molecules (modulators of WNT activity) enhance the hESC derivation and single-cell clonal expansion (Ai et al., 2020). These data suggest that the TGF-β/Activin/Nodal pathway is essential in human pluripotency during development, but in hESCs its activation is not enough, as it requires synergy with other pathways like FGF2 and WNT.

### FGF Pathway, the Dispensable Signaling for hPSCs

Exogenous FGF2 is enough to maintain pluripotency gene expression, promoting cell-cell adhesion and increasing survival (although not proliferation) in human pluripotent stem cells (hPSCs). The culture of hPSC (either on feeder layer or feeder-free conditions) dependent on FGF2 is known as the conventional condition. However, FGFR1, FGFR2, FGFR3, and FGFR4 expression has not been detected in epiblast; in contrast, FGFR1, FGFR2 and FGFR4 were enriched in PE (Niakan and Eggan, 2013; Kunath et al., 2014; Wamaitha et al., 2020). Furthermore, FGF2 is downregulated in epiblast compared with undifferentiated hESCs, which express the four FGF receptors and even FGF2 receptor isoforms, suggesting that the FGF2 dependence to support pluripotency is an aberrant condition in human pluripotency *in vitro*. However, exogenous FGF2 increases the phosphorylation of ERK1/2, AKT1/2/3, and GSK3α/β, suggesting downstream activation of MAPK and PI3K-Akt pathways and Wnt signaling regulation in hPSCs (Dvorak et al., 2005; Eiselleova et al., 2009; Ding et al., 2010). FGF receptor blockage with SU5402 or shRNA-mediated knockdown induces pluripotency marker downregulation and hESC differentiation, supporting the relevance of the FGF2 pathway to maintain the *in vitro* human pluripotency. LY294002 (inhibitor PI3K-Akt) administration significantly affects the hESC undifferentiated state compared with the ERK inhibition, suggesting that the principal downstream signaling cascade of FGF2 is PI3K/Akt rather than the MAPK pathway. Intriguingly, the EGF receptors (EGFR, ErbB2, and ErbB3), insulin receptor (INSR) and IGF receptor 1 (IGFR1) belong to the tyrosine kinase (RTK) family, which is highly phosphorylated in hESCs (Eiselleova et al., 2009). All these data suggest that alternative molecular pathways to FGFR activation can support human pluripotency and exist in the human epiblast.

### IGF Pathways and Crosstalk With AKT/mTOR

INSR and IGFR1 are highly expressed in the epiblast as compared with TE and PE in human embryos. As previously mentioned, hESCs have these receptors, and the addition of IGF1 increases their survival and growth, suggesting a novel role for the IGF pathway to regulate human pluripotency. Hence, a defined culture medium with only Activin and IGF1 is enough to derive hESCs (AI hESC) and reprogram hiPSC, maintain a similar expression of pluripotency-associated genes compared to the FGF2-dependent condition and retain their pluripotent potential to generate derivatives of the three germ layers, demonstrated through directed and spontaneous differentiation protocols (Wamaitha et al., 2020).

In the human blastocyst, treatment with IGF1 induces NANOG^+^ cell proliferation but does not induce changes in the number of SOX17^+^ cells, indicating an IGF1 effect exclusively on the epiblast lineage (Wamaitha et al., 2020). Interestingly, FGF2 treatment does not have any impact on the human embryo. Thus, the FGF2 exogenous dependence to maintain pluripotency in hPSCs could be an artifice condition, where the role of IGF signaling in *in vivo* human pluripotency is supplanted. Indeed, phosphorylated AKT-S473 and p70S6 kinase detection in human blastocysts (7 days IVF) would suggest activation of PI3K-AKT and mTOR (mammalian target of rapamycin) pathways as the IGF1 downstream effectors, which are also activated in hPSCs and are essential for their survival and pluripotency (Wamaitha et al., 2020). mTOR selective inhibition with rapamycin induces death cells in hESCs, while blocking PI3K through LY294002 decreases the expression of pluripotent markers such as OCT4 and TRA-1-60 (Ding et al., 2010; Yilmaz et al., 2018). These data suggest that, unlike the FGF pathway, TGF-β signaling and the crosstalk between IGF/PI3K-AKT/mTOR pathways are indispensable during early development and maintain hPSC identity.

### Modulation of the WNT Pathway in Human Pluripotency

The Wnt/ß-catenin pathway in synergy with LIF maintains mESCs self-renewal (Ogawa et al., 2006). However, the possible role of Wnt/ß-catenin in human pluripotency is contradictory. Its activation through GSK3 inhibition promotes self-renewal in hESC (Sato et al., 2004). Nonetheless, WNT signaling is not necessary for self-renewal and its activation induces differentiation (Dravid et al., 2005; Davidson et al., 2012). Thus, this suggests that the Wnt pathway promotes hESCs self-renewal or differentiation depending in the cellular location of ß-catenin: its retention in the cytoplasm by stabilizing AXIN2 supports the identity of the hESCs, but their nuclear translocation and interaction with TCF induces differentiation (Kim et al., 2013). ß-catenin also interacts with E-cadherin, α-catenin and actin in the cytoplasm, which would affect cell adhesion processes and signal transduction (Yamada et al., 2005).

This functional duality of the WNT signaling in human pluripotency could be explained by studies carried out under culture conditions where the hESCs are composed of heterogeneous populations. Indeed, the WNT pathway is decisive to the transition of pluripotent states. The diverse states of pluripotency that would reflect the progress of the epiblast during development will be discussed in the following sections.

## HPSC, Artifice or *In Vitro* Counterpart of the Human Epiblast?

**FIGURE 2:**
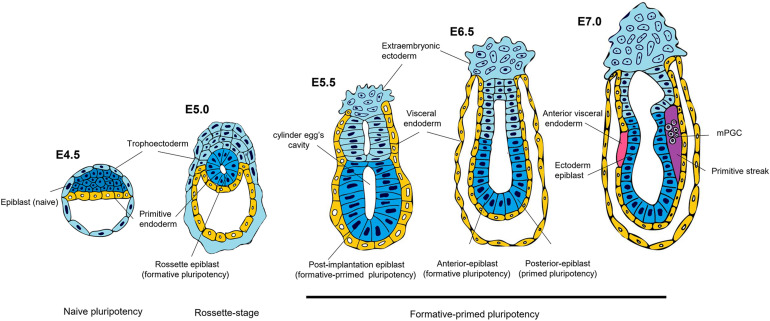
Spectrum of pluripotency in the mouse embryo. At 4.5 days post-fertilization (E4.5), the mouse epiblast has acquired a naïve state, the most sublime state of pluripotency. On E5.0 the blastocyst is implanted, where the epiblast forms a rosette structure. It has been proposed that this stage represents an intermediate pluripotency between the pre-implanted naïve and post-implanted primed states. In *in vitro* studies, this intermediate state has been described as formative pluripotency. On day 5.5 post-implantation, the formative-primed epiblast has a molecular and potential configuration different from that of the naïve state. On day 6.5, pluripotency can be divided into anterior and posterior regionalization of the epiblast. The anterior epiblast maintains a formative pluripotency, while the posterior epiblast acquires a primed pluripotency. The hypothesis proposes that on day 7.5, the posterior epiblast has a potential bias toward mesodermal precursors of the primitive streak, and the anterior epiblast acquires an ectoderm identity; its posteriorization is avoided by signals from the anterior visceral endoderm. Furthermore, mouse primordial germ cells (mPGCs) are specified in the posterior proximal epiblast from a pre-gastrulation subpopulation.

There are at least two types of pluripotency in the mouse (naïve and primed), which represent the pre- and postimplantation epiblast (preEPI and postEPI), respectively (Davidson et al., 2015; Avila-Gonzalez et al., 2016; Figure 2 and Table 1). Both states can be captured *in vitro* when the embryonic cells are adapted to grow in specific culture conditions. A defined medium supplemented with LIF, GSK3 inhibitor CHIR99021, and MEK inhibitor PD032 (2iLIF) is necessary to maintain the cells similar to those of the preEPI (Boroviak et al., 2014). On the other hand, the epiblast stem cells (EpiSCs) incubated with Activin A and FGF2 correspond to the *in vitro* counterpart of the postEPI (Brons et al., 2007). In a development context, the essence that distinguishes 2iL mESC from EpiSCs is that the first condition represents the “naïve ground state” of the preimplanted embryo, where embryonic cells do not have predefined programming but are rather a “blank slate” receiving the appropriate signals to start the specification and formation of lineage cells (Hackett and Surani, 2014). Therefore, 2iL mESC integrates efficiently into a preimplantation blastocyst, generating a chimera, while EpiSCs fail to integrate (Brons et al., 2007; Alexandrova et al., 2016).

**TABLE 1:** Pluripotent spectrum during the early developmental stages of mice.

Pluripotency spectrum in mouse				
	Naïve pluripotency	Intermediate pluripotency	Primed pluripotency	Regionalized formative-primed pluripotency (Peng)
Mouse embryo stage	E4.5 Preimplanted epiblast	E5.0-E5.5 Rosette epiblast stage	E5.5 Postimplanted epiblast	E6.0-6.5 Anterior epiblast (formative) Posterior epiblast (primed)
	2iL mESC (Boroviak et al., 2014; Boroviak and Nichols, 2017)	Rosette stern cells (Neagu et al., 2020)	EpiSC (Brons et al., 2007)	
	GSK3 inhibition ERK inhibition LIF(JAK/STAT3)	WNT inhibition ERK inhibition LIF(JAK/STAT3)	FGF Activin A	
*In vitro*			Formative stem cells (counterpart E5.5-6.0) (Kinoshita et al., 2021)	FTW-mESC (counterpartE5.5-6.0) (Yu et al., 2021a, b)
			Activin A WNT inhibition Retinoic acid (agonist)	FGF2 TGFβ-1 WNT activation

Regarding hESC, single-cell RNA sequencing demonstrated that conventional hESC and human epiblast have different transcriptomes with 1498 genes showing differential expression. Interestingly, the cells during the derivation method drastically change their gene expression; there was a downregulation in genes related to human epiblast such as NANOG, GDF3, ESRRB, DPPA5, IGF1, KLF4, and KLF17, with increased pluripotency-related (ZIC2, ZIC3, ID1, ID2, and ID3) and members of FGF and WNT signaling pathway genes. Since genes involved in the FGF pathway (FGF2, FGF5, FGFR1, and FGFR4) are also enriched in EpiSC, this was one of the first studies to suggest that conventional hESC are more similar to EpiSC than mESC, and therefore, to primed pluripotency (Yan et al., 2013).

Similarly, another single-cell RNA sequencing revealed that hESCs retain human-specific epiblast-enriched genes (KLF17 and the Nodal pathway) as well as a high expression of genes related to the FGF and WNT pathways (Blakeley et al., 2015). Additionally, principal component analysis has revealed that the presumptive transcriptional state of conventional hESCs, which do not cluster with the human blastocyst ICM and E5.0-E7.0 human epiblast, significantly resemble primed EpiSCs and the E5.5 mouse postEPI (Yang et al., 2019).

On the other hand, regarding the capture of the naïve state in human, diverse groups have reported obtaining this presumptive state using different cocktails of growth factors and small molecules (Avila-Gonzalez et al., 2016); but there is no consensus to establish the conditions to capture the *in vitro* human pluripotency that represents the counterpart of the preimplantation embryo. Several protocols report obtaining hPSC that express components of the circuitry which supports the naïve pluripotency of mouse epiblast and mESCs (Gafni et al., 2013; Takashima et al., 2014; Theunissen et al., 2014; Guo et al., 2016). However, naïve hPSC expressed human epiblast-enriched genes (DPPA3, DPPA5), which makes it more similar to the human epiblast than the primed conditions. They also overexpress pathways (FGF4) that are not represented in the human epiblast (Blakeley et al., 2015). According to the mESC 2iL potential, human naïve lines should be more reliable to recapitulate a differentiation process than conventional hESCs. However, presumptive naïve hESCs are deficient in differentiating at least into functional cells of cardiac and neuronal lineages, contrasting with primed hESCs (Warrier et al., 2017). Therefore, although conventional and naïve hPSCs share human epiblast molecular characteristics, the bizarre enrichment of signaling pathways and pluripotency-related genes might be an artifice due to the “unnatural” adaptation to proliferate in culture. Another possibility is that the current conditions to maintain hESCs derive a heterogeneous population mixing several “subtypes” of pluripotency.

Indeed, the failure to capture the human primed and naïve pluripotency could be explained by the broad pluripotency spectrum in primates. Delineation of the early developmental stages in the cynomolgus (cyno) monkeys by single-cell RNA seq, demonstrated that the epiblast segregates into preEPI, as well as early and late postimplantations (postE-EPI and postL-EPI). Thus, compared with cyno embryo cells, conventional hESCs have a higher correlation with the postL-EPI, compared to the naïve conditions with the preEPI (Nakamura et al., 2016; Table 2). Another series of studies in *in vitro* cyno embryo confirms that the later-stage cyno epiblast cells express genes related to primed pluripotency (ZIC2, ID1, and FGFR1) through single-cell seq (Niu et al., 2019), just like conventional hESC.

**TABLE 2:** Presumptive human pluripotent spectrum during the human development and its analogy with primate cyno.

Pluripotency spectrum in human						
	Naïve pluripotency		Intermediate pluripotency (formative)	Primed pluripotency	Regionalized formative-primed pluripotency	
Human embryo stage (Xiang et al., 2020)	ICM (6–7 days IVF)	Preimplanted epiblast (6–9 IVF)		Postimplanted-epiblast (8–12 days IVF)	Primitive streak-epiblast (12–14 days IVF)	
Cyno embryo stage (Nakamura et al., 2016)		Preimplanted epiblast (E6-9)		Postimplanted early and late epiblast (E12 E16)		Gastrulating cells (> E14)
*In vitro*	Expanded potential stem cells (Gao et al., 2019)	Naïve hPSC		Conventional hPSC (Takashima et al., 2014)	Region selective hPSC (Wu et al., 2015)	Naïve hPSC (Gafni et al., 2013)
	LIF, Activin A, ascorbic acid and inhibitors of WNT pathway/SRC/GSK3	5iLA hPSC (Theunissen et al., 2014) (LIF, Activin A, GSK3/ERK2/BRAF/ROCK/SRC inhibitors)		FGF2 Activin A/TGF-β	FGF2 WNT inhibition	LIF(JAK/STAT3). inhibitors of GSK3, ERK, JNK, PKC and ROCK, low doses of FGF2 and TGF-β1
		2iL+Gö hPSC (Takashima et al., 2014; Guo et al., 2021) (LIF, GSK3/ERK/PKC inhibitors	FTW-hPSC (counterpart human epiblast E8) (Yu et al., 2021a, b)	Formative hPSC (counterpart human epiblast E10-12) (Kinoshita et al., 2021)		
			FGF2, TGβ-1 WNT activation	Activin A, WNT inhibition and retinoic acid (agonist)		
	Mesoderm like cells hPSC (germinal pluripotency) (Chen et al., 2019) Resetting conventional hPSC with Activin A & GSK3 inhibitor					

Human epiblast embryos can be classified based on their transcriptomic profile into four stages: ICM (6–7 days IVF), preEPI (6–9 days IVF), postEPI (8–12 days IVF), and primitive streak-epiblast (PS-Epi) (12–14 days IVF) (Xiang et al., 2020). When hPSCs are compared to these transcriptomic profiles, the principal component analysis indicates that the naïve lines are clustered with PreEPI, while the primed lines are adjacent to postEPI, however, distributed toward the PS-Epi (Kinoshita et al., 2021). Therefore, conditions alternative to naïve and primed have been described in the literature where hESCs can recapitulate some fraction of this broad pluripotent spectrum of the human epiblast.

Finally, hPSC cultured with XAV939 (WNT inhibitor), Activin A and FGF2 (XAF) acquire the capacitance to substantial multi-differentiation through a transition period that might represent the epiblast progression during implantation, being a defined state different from naïve and primed (Rostovskaya et al., 2019). This intermediate phase, as well as other pluripotent substates, are described in the next section.

## The Spectrum of Pluripotency in Mammals

### Rossette-Stage Pluripotency in Mouse

A recent study has characterized an intermediate stage in mouse epiblast different from the naïve and primed stages. Before implantation (E4.5), the epiblast is identified by expressing naïve associated genes such as Klf4. Around E5.0, it develops a “rosette,” displaying nascent lumen at E5.5 (lumenogenesis), which will be the egg cylinder cavity (Figure 2). At the “rosette” stage, the cells are still Klf4^+^; however, they express Otx2 (primed pluripotency-associated gene), absent at E4.5. During the “lumen” stage, the epiblast maintains the Otx2 expression and colocalizes with Oct6 (post-implanted epiblast marker). There are defined signaling pathways to regulate this naïve-rosette-lumen transition in the mouse embryos. Treatment with IWP2, a Wnt inhibitor, prevents rosette formation. In contrast, an ERK inhibitor arrests the rosette cells and avoids lumenogenesis, demonstrating that downregulation of the Wnt pathway is necessary for the epiblast’s transition from the naïve stage into the rosette stage, while ERK signaling activation enhances the progression into the lumen stage. Therefore, cells in the rosette stage, named rosette-like stem cells (RSCs), can be captured *in vitro* with a defined cocktail (LIF/Wnt inhibitor IWP-2/MEK inhibitor PD032) (Table 1). They are KLF4^+^ /OTX2^+^ /OCT6- similar to the epiblast at E5.0 (rosette stage) and have a molecular profile different from 2iL mESCs (naïve) and primed epiblast-like cells (EpiLCs) and display an epigenetic landscape with a distinctive feature that possesses twice the number of bivalent genes. Also, they do not have a downregulation in naïve genes or present inactivation of the X chromosome. These data demonstrate the capture of a transition between naïve and primed states that depends on WNT and MEK/ERK signaling pathway inhibition for maintenance (Neagu et al., 2020).

### Formative Pluripotency in Mouse and Human

Before the epiblast rosette stage was characterized, it had been suggested that pluripotency could have an intermediate state in addition to naïve and primed. Instead of being static and rigidly delimited, the intermediate state would be a continuum where the embryonic cells dynamically change their molecular, transcriptional and epigenetic profiles in response to autocrine signals coming from the cell-cell interaction or the extracellular environment to progress through development. According to this hypothesis, naïve cells transition into a formative phase and acquire competence for germ lineage induction and somatic lineage specification before entering primed pluripotency (Smith, 2017). The first evidence that pointed out the difference of formative from naïve and primed stages were carried out in mESCs. Thus, 2i medium was withdrawn and mESCs were monitored as they exited the naïve state. When the naïve factors disappeared, the expression of post-implantation molecules [Pou3f1 (Oct6), Otx2, Sox3, Sall2, and Fgf5] signaling pathways only present in the primed post-implantation period (ERK pathway), were observed. Also, *de novo* methylation levels associated with the expression of Dnmt3a/3b increased, mitochondrial respiration decreased and JAK/STAT, WNT, and mTOR signaling were downregulated (Kalkan et al., 2017).

Because WNT inhibition is important for the transition from the naïve to the rosette stage in mice, similar strategies have been designed to block this path to capture both mouse and human formative pluripotency *in vitro*. Activin A in a low concentration, XAV939 (WNT inhibitor) and an inverse agonist of the retinoic acid receptor (A_low_XR) can derive mouse lines showing a correlation with pre-PS-Epi at stage E5.5-6.0 (Table 1). The embryonic cells cultured in A_low_XR medium were designed as formative stem (FS) cells because they are more competitive for primordial germ cell (PGC) induction than EpiSC and naïve mESC; corresponding to a pluripotent formative state according to Austin Smith’s hypothesis. Similarly, naïve hPSC treated with A_low_XR medium to obtain FS-like cells have a molecular profile similar to the human epiblast at 10–12 days IVF embryo (Kinoshita et al., 2021; Table 2).

Another study derived mouse and human intermediate PSC with activated FGF2, TGF-β, and WNT pathways (FTW-mESC and FTW-hPSC, respectively, Tables 1, 2). This experimental condition activates the WNT pathway (using high concentrations of WNT3A or GSK3 inhibitors such as SB216763 and CHIR99021). Intriguingly, comparable to FS cells, the FTW-mESC expression profile clusters are close to the mouse epiblast E5.5-6.0 and the cells are highly competent to form PGCs. At the same time, the FTW-hPSC transcriptome correlates to the human epiblast 8 days IVF embryo (Yu et al., 2021b).

On the other hand, after 2i withdrawal, a gene-specific network (including Klf5, Nr0b1, Nr5a2, and Pdgfa) related to naïve pluripotency was detected in mESCs, which is necessary for exiting from the naïve state and transitioning to the formative stage. Interestingly, this network was found not only in mouse embryos in the implantation stage (E5.5) but also in macaque postEPI and capacitance hPSCs (Lackner et al., 2021). These data suggest that conversion from the naïve to the formative phase is conserved in primates through a gene network mechanism.

### Diversification of Postimplantation Pluripotency in Primates

In the mouse, the first pluripotent cells (Nanog^+^) appear at E3.5 in the ICM, differentiate into the epiblast and remain pluripotent after implantation at E5.5. However, the molecular network that maintains their identity is lost at around E7.5-8.0, once gastrulation begins at E6.5. In contrast with the mouse, whose pluripotency declines approximately 2 days after implantation, transcriptomic studies for *in vivo* and *in vitro* embryos in cyno monkeys have suggested that pluripotency extends far beyond post implantation until E16 or 16–20 IVF days (Nakamura et al., 2016; Ma et al., 2019; Niu et al., 2019). Thus, the temporal period of pluripotency is more extended in primates.

During post implantation, several critical outcomes take place: the yolk sac cavity develops, the amniotic epithelium arises (and lumenogenesis of amnion cavity occurs) and the EXM as well embryonic lineages (PGCs) and the primitive streak (PS) precursor specification take place (Niu et al., 2019). Moreover, the emergence of EXM and amniotic epithelium specification during implantation is a punctual difference that primates exhibit compared to murine (Boroviak and Nichols, 2017; Ross and Boroviak, 2020).

The amniotic epithelium is the innermost membrane that surrounds and protects the embryo during development, while the chorion and allantoides support the transport of nutrients and vascularization derived from EXM. Intriguingly, the amniotic epithelium derives from a subpopulation of the epiblast. In contrast, the origin of the EXM still is unknown, although it has been suggested that it may arise from the PE (hypoblast) or epiblast (Niu et al., 2019; Ross and Boroviak, 2020). It is unknown whether PGCs and precursors of PS derive from primed substates or an intermediate phase between naïve and primed pluripotency, although it has been proposed that they emerge from the epiblast and/or amnion (Sasaki et al., 2016; Ma et al., 2019; Niu et al., 2019) or from an intermediate pluripotent subpopulation (named germinal pluripotency; Chen et al., 2019). The cellular interactions between epiblast and the other extraembryonic lineages (at least the TE, PE and EXM) may be predicted through receptor-ligand database analyses (Niu et al., 2019). However, this has not been demonstrated experimentally in primate embryos. Thus, the multiple lineage interactions with the epiblast could influence its progression until the disappearance of its pluripotency, which would delimit other pluripotency subtypes still not characterized in primate embryos.

### Germinal Pluripotency’ in Primates

One of the substates could be the putative “germline pluripotency” to specify germ cells. PGCs present pluripotency-related molecules and arise from a pluripotent source prior to gastrulation. The consensus to differentiate human PGCs (hPGCs) from conventional primed hPSCs involves Activin A and CHIR99021 exposure to induce incipient mesoderm-like cells (iMeLCs). In this transitory phase, embryonic cells form and then become hPGCs (Sasaki et al., 2015). Surprisingly, iMeLC express naïve and primed markers besides presenting an intermediate state. Upon identifying the cyno embryonic counterpart of the iMeLCs, researchers found that conventional primed hESCs were more similar to the postE-EPI and postL-EPI, whereas iMeLCs were consistent with ICM and preEPI as well as with postE-EPI and postL-EPI from cyno embryos. These data suggest that iMeLCs might recapitulate a transitory phase during implantation, where germline specification occurs, so it would be named “germinal pluripotency” (Chen et al., 2019; Table 2).

Interestingly, the signaling and molecular mechanisms that orchestrate germline specification are different in humans and mouse models (Irie et al., 2015; Kojima et al., 2017). Therefore, this “germinal pluripotency” would be primate-spectrum exclusive. Its signature gene expression (TFAP2A, EOMES GATA3, and BRACHYURY) is related to gastrulation and the amniotic epithelium, which is also derived from the human epiblast in the peri-implantation stage (Chen et al., 2019). Hence, once it leaves the naïve stage, does the human epiblast diverge into different postimplantation pluripotent subpopulations? Or is it competent for other lineages in addition to the three embryonic layers, such as the amnion and the germline?

### Pluripotency Biased Toward Embryonic Lineages in hPSCs

hPSCs have subpopulations with a bias toward a particular lineage differentiation. Although they retain their high capacity for self-renewal and pluripotent potential, these subpopulations express specific lineage markers that allow them to have a strong differentiation bias. The GATA6^+^ hPSC subpopulation has a greater predisposition to differentiate into the endoderm lineage than GATA6^–^ hPSCs in conventional conditions (KOSR/FGF on feeder layer, or E8 medium on feeder-free culture; Allison et al., 2018). On the other hand, E8 medium supplemented with BSA, cholesterol, CHIRON, IWP-2, and lysophophatidic acid promotes an enrichment of MIXL1^+^ hPSCs, which exhibit a mesodermal lineage-bias (Stavish et al., 2020). Besides, the culture medium may do more than induce heterogeneity in the hPSC differentiation potential. The change of feeder-free substrate from Vitronectin to Matrigel may influence two substates, identified by the VIMENTIN^−^ or VIMENTIN^+^ population. The first type on Matrigel has an expression profile of genes involved in neuronal function (COL9A2, DGKI, GBX2, KIF26B, MARCH1, PLXNA4, SLC24A4, TLR4, and ZHX3) and a potential bias toward ectoderm lineage, in contrast with the second type cultured on Vitronectin (Mawaribuchi et al., 2019).

In light of the hPSC substrates *in vitro*, there would be primed subpopulations biased toward a specific embryonic layer in the post implanted human embryo. These substrates have not been characterized to complete the landscape of primed pluripotency, considering notable differences in human and other primates vs. mice in the context of their extraembryonic lineage interactions. In summary, the rosette stage in the mouse embryo, transitory *in vitro* iMeLCs (germinal pluripotency) in humans, and formative pluripotency in both species are clues to elucidate the transition between naïve and primed pluripotency.

### Extra-Embryonic Potential of Naïve hPSC

It has recently been reported that hPSC can differentiate into both embryonic (epiblast) and extraembryonic (trophoblast and hypoblast) lineages. Therefore, it is possible to establish human pluripotent cells with the ability to differentiate into TE-like cells (named human expanded potential stem cells, hEPSC) with LIF, Activin A, ascorbic acid and inhibitors of both WNT pathway and SRCs and GSK3 kinases (Gao et al., 2019; Table 2).

Naïve hPSC-derived cells may express trophoblast markers (KRT7, TFAP2C, TEAD4, and GATA3) through spontaneous differentiation (embryonic body formation), directed differentiation to generate TE-like cells and derivation of human trophoblast stem cells (hTSC) lines (naïve-hTSC). Indeed, both TE-like cells and naïve-hTSC committed to extravillous trophoblast (EVT) and syncytiotrophoblast (STB) cells. Intriguingly, 2iL mESC cannot differentiate into TE lineages without genetic and epigenetic manipulations, while for primed hESC, its yield is meager as compared to the naïve condition to generate the trophoblast cells (KRT7, TFAP2C, TEAD4, and GATA3) (Dong et al., 2020; Guo et al., 2021; Io et al., 2021).

On the other hand, in naïve hPSC, Nodal, Wnt, and LIF activation pathways induce PE markers such as PDGFRA, GATA6, and NID2. These PE-like cells can be expanded *in vitro* for several passages (Linneberg-Agerholm et al., 2019). Hence naïve hPSC, unlike naïve mESC, and primed hPSC, are competent to differentiate into extraembryonic tissues, so the restriction of lineage paradigm would not be implemented in human pluripotency.

Indeed, the exceptional plasticity of human pluripotency is illustrated by recent reports, which generates blastocyst-like structures (blastoids) (Liu et al., 2021; Yu et al., 2021a). The blastoid-derived hPSC model would allow to study the dynamics of the pluripotent spectrum from the preimplantation period (specification of the epiblast, the plasticity of the naïve pluripotency, and changes in the epiblast during the implantation process, among others) and recognize their differences and similarities vs. the most studied pluripotency model of the mouse epiblast.

## Spatial Regionalization of Pluripotency

Pluripotency not only progresses through a time window but is also regionalized. Its transcriptome’s dynamic expression throughout pre- and post-implanted mouse embryo showed that naïve pluripotency-related genes disappeared upon epiblast implantation, giving rise to a set of genes related to formative pluripotency at E5.5. As development continues, the first epiblast regionalization occurs, dividing into anterior and posterior regions at E6.5. Interestingly, the transcription of the formative genes is retained in the anterior epiblast, where the ectoderm specification begins at E7.0-7.5. In contrast, the posterior epiblast will produce a PS with a transcriptome profile related to primed pluripotency at E6.5 and decline its expression until E7.5 (Figure 2; Peng et al., 2019). These data suggest that pluripotency has not only a temporal progression component but also a spatial division. Indeed, embryonic cells acquire a regional identity before inducing their differentiation into either anterior or posterior structures (Metzis et al., 2018).

Interestingly, this spatial pluripotency has been described in humans. Belmonte’s laboratory discovered that hPSCs derived or maintained with FGF2 plus IWR1 displayed an alternative molecular profile different from conventional primed pluripotency (Table 2). According to their functional potential, these cells were denominated region-selective PSC (rsPSC) because they contribute to chimera formation in non-intact E7.5 mouse embryos only in the posterior region (Wu et al., 2015). rsPSC simulates *in vitro* features of the posterior epiblast and supports the existence of regional pluripotency in humans.

On the other hand, the blastoids which are composed of cells similar in morphology and transcriptomic profile to the three blastocyst lineages (TE, epiblast, and hypoblast), as well as with a specific spatial positioning that allowed the formation of a blastocele-like cavity (Liu et al., 2021; Yu et al., 2021a); corroborate the plasticity of hPSC to differentiate into extra-embryonic lineages, both from the first and second lineage specification of the blastocyst (Gao et al., 2019; Linneberg-Agerholm et al., 2019; Dong et al., 2020; Guo et al., 2021; Io et al., 2021). This expanded or bipotent potential of hPSCs reflects that the regional spectrum of human pluripotency might not only diverge during the post implantation period. Also, in the preimplantation stage there would be pluripotent subpopulations, one already committed to the epiblast lineage, and others with plastic capacity to give rise to the hypoblast or to retain the potential toward the TE.

Regarding the post implantation period pioneering studies demonstrated that hPSC self-organization resembles that of the embryonic sac, consisting of a cyst of asymmetric cells separated by a lumen that corresponds to the amniotic cavity. hPSCs are subjected to BMP4 signaling through a dorsal-ventral gradient and differentiate into amniotic ectoderm-like cells (AELCs) from the dorsal pole to achieve this lumenogenesis. Depending on the signals that hPSCs receive on the ventral side, embryo-like cysts acquire an anterior or posterior identity. In the posteriorized sacs, precursor cells of the PS and germline marker expression are observed at the ventral pole.

The anteriorized structure consists of AELCs on the dorsal side, separated by the lumen, and epiblast-like cells (EPILC) on the ventral part. EPILC transition to gastruloid-like cells is suppressed with inhibitors of the WNT and BMP pathways (Zheng et al., 2019). This suggests that once the amniotic cavity is formed, pluripotency is restricted in the anterior part of the embryo sac, since the PS and germ cell specification begins in the posterior embryo. Furthermore, this primed pluripotency subpopulation sheltered in the anterior region of the embryo coexists and interacts with the amniotic epithelium. Therefore, amniogenesis could be relevant for the developing embryo and notably for anteriorized structures.

## The Amnion Arises From the Epiblast

**FIGURE 3:**
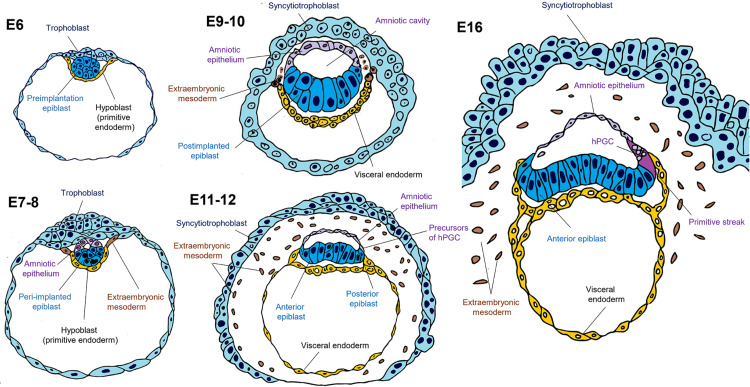
Spectrum of pluripotency in the human embryo. On embryonic day 6 (E6), the human blastocyst is pre-implanted, with probable naïve pluripotency. During implantation (E7-8), two lineages emerge in human embryos: the extra-embryonic mesoderm from the visceral endoderm (hypoblast) and the amniotic epithelium from the epiblast. On days 9–10, the amniotic cavity arises, while the post-implanted epiblast still expresses genes related to pluripotency but is no longer in the naïve state. It has been suggested that human primordial germ cell (hPGC) specification occurs at around E11-12, but also both lineages can arise from the same population that possesses germinal pluripotency. It is likely that the human epiblast, similar to the mouse, acquires an anterior-posterior regionalization with different subtypes of pluripotency biased toward specific lineages. On day 16, the onset of human gastrulation is preceded by the appearance of the primitive streak in the posterior region of the epiblast.

Decades ago, in the absence of experimental evidence, the amnion’s origin from the epiblast was deduced from observations of histological sections of primate embryos. At around E7.0-9.0 (Carnegie stage 5a and 5b), amniogenesis begins in the human embryo, with a distinguished amniotic cavity at E10.0 (Carnegie stage 5c) (Figure 3; Hertig et al., 1956). Similarly, in the macaque embryo, the amniotic cavity lumen appears at E10.0 and is fully defined at E10.5-11.0 (Enders et al., 1986).

In 2016, two research groups independently reported for the first time the *in vitro* culture of human embryos to study their postimplantation development. Between days 7 and 8 after IVF, the embryos were adhered to a plate and implantation was simulated for up to 14 days. The research groups observed at 9–10 days that a subset of epiblast cells acquired an apical-basal polarization and organized radially around a lumen that corresponds to the nascent amniotic cavity (Deglincerti et al., 2016; Shahbazi et al., 2016).

On the other hand, the emergence of the amniotic cavity and the dissection of two OCT4^+^ populations, one columnar near the anterior visceral endoderm (AVE) and the other squamous on the TE side has been reported during *in vitro* culture of macaque embryos at 11–12 days IVF (Ma et al., 2019; Niu et al., 2019). Indeed, the single cell RNA-seq analysis carried out from recent 3D culture human embryo experiments confirmed that on 12–14 days IVF the amniotic epithelium and the epiblast are already two separate populations, each with a unique transcriptomic profile (Xiang et al., 2020).

On the other hand, it has been demonstrated that human amnion derives from the epiblast using a model of 3D self-organization of hPSCs. Thus, hPSCs were grown in a biomaterial system to mimic the implantation niche and formed a cyst constituted by a columnar epithelium (the presumptive epiblast) and squamous cells, which were considered AELCs (Shao et al., 2017; Zheng et al., 2019).

These elegant experiments confirm the hypothesis that the amniotic epithelium is derived from the human and cyno epiblast. However, the mechanisms involved in specifying the epiblast toward the amnion are still unknown.

Messmer et al. detected a subpopulation with specific genes (such as VGLL1, XAGE2, GATA3, ERP27, NR2F2, VTCN1, ODAM, HSD3B1, P2RY6, and EGFR) not expressed in naïve or primed hPSCs (Messmer et al., 2019). Besides, several of these genes are enriched in extraembryonic tissues (trophoblastic and endothelial cells from placenta) and upregulated in the amnion-like cells derived from hPSCs (VGLL1, XAGE2, GATA3, HSD3B1, and P2RY6) (Shao et al., 2017). Therefore, these “intermediate genes” sign a formative or primed pluripotent subpopulation that will originate the amnion.

On the other hand, experiments with cyno monkey embryos indicate that PGC specification is at around E11-12 days when the amnion is a separate lineage from the epiblast. Thus, it has been suggested that primate PGCs are specified from the nascent amnion (Sasaki et al., 2016; Niu et al., 2019). However, it is also possible that both amnion and PGCs derive from the same pluripotent population that later diverges (Figure 3; for PGCs, this subpopulation is described as “germinal pluripotency” in Chen et al., 2019). These data suggest that the epiblast subpopulation where amniogenesis occurs would be an intermediate state between naïve or primed pluripotency or part of the spectrum of primed epiblast amnion that precedes the PGCs or both share a common pluripotent lineage.

## Possible Role of the Amnion in the Anterior-Posterior Regionalization

During cyno development, a disc-shaped embryo, composed of the columnar epithelium (epiblast), amniotic membrane, and yolk sac, and surrounded by the extraembryonic mesenchyme, is settled at E14. In this stage, the epiblast maintains the expression of a cluster of factors related to “pluripotent stemness” (POU5F1, NANOG, SOX2, and PRDM14) before gastrulation at E16. Still, the postL-EPI express genes associated with “neuron development” (Nakamura et al., 2016; Ma et al., 2019). In human embryo culture, it was also detected that the population considered postimplantation epiblast, apart from still expressing the transcriptional program related to pluripotency, begins to express genes involved with neural development (Xiang et al., 2020).

Therefore, in primates, neural induction occurs in the anterior region, as described in mice and chick embryos. The AVE is in contact with the most anterior part of epiblasts in mouse, chick and non-human primate. DKK1 and CER1, antagonists of WNT and BMP/TGF-β signaling pathways, respectively, are expressed in AVE at E11 during the development of cyno (Sasaki et al., 2016). Interestingly, WNT and BMP pathways are required to induce the formation of the PS in the posterior region of the epiblast in several species.

**FIGURE 4:**
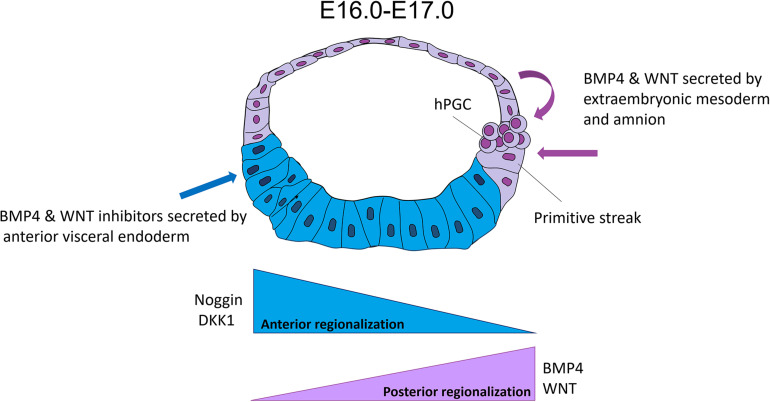
Hypothesis of the human epiblast regionalization. This hypothesis suggested that human pluripotency is regionalized into anterior and posterior. The posterior subpopulation has a differentiation bias toward mesoderm precursors to initiate gastrulation at E16, probably because of signals (WNT and BMP4) from the extraembryonic mesoderm and amniotic epithelium. On the other hand, the anterior region is still pluripotent and prevents its posteriorization by signals (negative regulators of BMP4 and WNT such as CER1 and DKK1, respectively) from the anterior visceral endoderm (AVE).

Thus, in human and non-human primates, the AVE would avoid the “posterization” of the anterior epiblast through WNT and BMP/TGF-β path inhibitors (Figure 4).

The amnion express components of WNT (WNT3, WNT6, and AXIN), BMP4/TGF-β (BMP4, ID2, and MSX2) and PI3K/Akt signaling pathways (Sasaki et al., 2016; Niu et al., 2019; Yang et al., 2020) during the cyno embryo development. The BMP4 decreased by the loss of ISL1 in the amnion induces a failure in the mesoderm formation of the posterior epiblast in this species (Yang et al., 2020). The amnion may also have an essential role in the PGC specification via BMP4 and WNT3 (Sasaki et al., 2016). All these data suggest that the amnion would have a role as an extraembryonic lineage and promotes the pattern of the anterior-posterior regionalization in epiblasts, in addition to the AVE at least in human and non-human primates (Figure 4).

Elucidating amnion ontogeny is relevant for embryonic development to complete the pluripotent spectrum of epiblast and its possible participation in the specification of lineages, such as PGC and the posterior mesoderm, at least in primates. As mentioned, hPSCs can self-organize into two embryonic like-sacs (ELS) with anterior (anterior-ELS) and posterior identity (posterior-ELS). Interestingly, AELCs are present in both cysts after an exposure to BMP4. The posterior-ELS comprises AELC in the dorsal part, whereas PS-like cells are found in the ventral zone. In accordance, hPSC co-culture with amniotic-like cells promotes their exit from pluripotency and begins to express PS markers (Zheng et al., 2019).

Likewise, in the anterior-ELS model, inhibition of WNT and BMP4 signaling paths avoid hPSC differentiation into posterior PS-like, maintained EPILC, where they coexist with the AELC. In addition, hESCs can be derived on a feeder layer of human amniotic epithelial cells, maintaining their expression of pluripotency-related markers and its functional potential demonstrated by the formation of embryoid bodies and teratomas (Avila-Gonzalez et al., 2015). Therefore, co-culture of isolated amniotic epithelial cells or AELC derived from hPSCs with embryonic cells is the experimental counterpart evidencing that there is a period in which the amniotic epithelium interacts with the epiblast, so it could have a role in the anterior-posterior regionalization such as the AVE.

The “default neural” model in developmental biology states that epiblast cells, in the absence of signaling for their fate, derivate ectoderm tissue (Munoz-Sanjuan and Brivanlou, 2002). However, before neural induction, the cells acquire a regional identity, which would provide the instructions and signals to establish the anterior-posterior pattern of the neuroectoderm derivatives (Metzis et al., 2018). We propose that the amniotic epithelium’s interaction is relevant for the differentiation of epiblast toward the anterior neuroectoderm fate. Indeed, the neuroectoderm is exposed to the amniotic fluid at E8.5 in the mouse embryo. Once the neural tube is closed, the amniotic fluid that remains inside the lumen would be the original cerebrospinal fluid (Chau et al., 2015). Therefore, amniotic fluid directly connects with the neuroepithelium, probably regulating embryonic neurogenesis and neural tube patterning.

As recent evidence supports the importance of the amnion and other extraembryonic lineages in human embryogenesis are two aspects where the interaction of the amnion with embryonic cells needed be studied. First, to analyze the co-culture of the amniotic-like cells with the hPSCs to simulate the peri-implantation period. With this model, to learn whether amnion specification is derived from an intermediate state between naïve and primed or shares the same origin subpopulation as the germline. In this way, studying amniogenesis would complete the spatiotemporal spectrum of human pluripotency. Second, to establish a model based on hPSC self-organization that contains two pluripotent subpopulations coexisting in the same ELS: one subpopulation with anterior fate starts neural induction, while the posterior subpopulation specifies into the mesoderm-PS. This model would also include a third group, AELC, to study their secreted factors or physical interaction with the subpopulations to promote neural induction and gastrulation.

Therefore, it can be inferred that state-of-the-art synthetic biology including the study of other non-embryonic lineages is relevant for the biology of human development and reproduction, fetal reprogramming and regenerative medicine.

## Author Contributions

DÁ-G, WP, GG-L, AM-H, ND-M and ND wrote sections of the manuscript. All authors contributed to manuscript revision, read, and approved the submitted version.

## Conflict of Interest

The authors declare that the research was conducted in the absence of any commercial or financial relationships that could be construed as a potential conflict of interest.
